# Travel Time Estimation Using Freeway Point Detector Data Based on Evolving Fuzzy Neural Inference System

**DOI:** 10.1371/journal.pone.0147263

**Published:** 2016-02-01

**Authors:** Jinjun Tang, Yajie Zou, John Ash, Shen Zhang, Fang Liu, Yinhai Wang

**Affiliations:** 1 School of Transportation Science and Engineering, Harbin Institute of Technology, Harbin 150001, China; 2 Department of Civil and Environmental Engineering, University of Washington, Seattle, WA 98195–2700, United States of America; 3 Key Laboratory of Road and Traffic Engineering of Ministry of Education, Tongji University, Shanghai 201804, China; 4 School of Energy and Transportation Engineering, Inner Mongolia Agricultural University, Hohhot 010018, China; Jiangnan University, CHINA

## Abstract

Travel time is an important measurement used to evaluate the extent of congestion within road networks. This paper presents a new method to estimate the travel time based on an evolving fuzzy neural inference system. The input variables in the system are traffic flow data (volume, occupancy, and speed) collected from loop detectors located at points both upstream and downstream of a given link, and the output variable is the link travel time. A first order Takagi-Sugeno fuzzy rule set is used to complete the inference. For training the evolving fuzzy neural network (EFNN), two learning processes are proposed: (1) a K-means method is employed to partition input samples into different clusters, and a Gaussian fuzzy membership function is designed for each cluster to measure the membership degree of samples to the cluster centers. As the number of input samples increases, the cluster centers are modified and membership functions are also updated; (2) a weighted recursive least squares estimator is used to optimize the parameters of the linear functions in the Takagi-Sugeno type fuzzy rules. Testing datasets consisting of actual and simulated data are used to test the proposed method. Three common criteria including mean absolute error (MAE), root mean square error (RMSE), and mean absolute relative error (MARE) are utilized to evaluate the estimation performance. Estimation results demonstrate the accuracy and effectiveness of the EFNN method through comparison with existing methods including: multiple linear regression (MLR), instantaneous model (IM), linear model (LM), neural network (NN), and cumulative plots (CP).

## Introduction

Travel time is defined as the time to traverse a route between a specified origin and destination. It is an important performance measure for road users and transportation managers alike as it is easily identified and understood by both groups. In current networks that employ advanced traffic management systems (ATMS), travel time not only reflects the traffic conditions of road network, but also affects the drivers’ route planning. Delays in travel time lead to increases in trip costs, vehicle emissions, and energy consumption. Thus, it is beneficial and challenging for using the travel time as an effective index to take measures on traffic congestion.

Methods for collecting travel time data can be generally divided into two categories. The first category involves direct approaches including the use of probe vehicles equipped with global positioning systems (GPS) [1–5], automatic vehicle identification (AVI) systems [6–8], Bluetooth (BT) devices [9–12]. Travel time data can be quickly collected via these approaches, but sample size and equipment coverage rate will impact the reliability of the analysis. For the GPS sensors, in order to collect travel time data accurately and detect fluctuations in measurements over time with probe sensors, the minimum number of the probe vehicles is required to be larger than two percent of the traffic volume [13–15]. Further, the periods of time over which the probe sensors collect data are required to be longer than a threshold [16–17]. For travel time collection methods that make use of AVI and BT technologies, obtaining the necessary level of equipment coverage is the main problem due to high equipment costs. Thus, in scenarios without extensive detector coverage where information is collected only along primary facilities (e.g., main roads and intersections), it is difficult to obtain reliable results for the whole network based on travel time.

The second category of methods for collecting travel time data involves an indirect approach in which loop detectors are the most commonly used equipment. Compare with the direct travel time estimation technologies, loop detectors have following several merits for estimating travel time: (1) high coverage rate, with its high distribution density, the traffic flow data collection and analysis in freeway network mainly depends on the loop infrastructure; (2) low cost, compare with GPS, AVI and BT, the cost of loop detectors is relatively low; (3) stable working state, GPS, AVI and BT are liable to be affected by weather and finally result in missing or inaccurate measurements. Loop detectors can work normally even in harsh circumstance; (4) abundant data sources, loop detectors can provide traffic flow data at different time interval in a whole day. Due to their advantages, many scholars and engineers focus on using data from loop detectors to estimate the real-time traffic states [18–23]. Currently, indirect methods to estimate travel time fall into four main classes: speed-based estimation models, cumulative plot based methods, regression models, and artificial intelligence, all of which are elaborated on in the following.

1. Speed-based estimation models, such as those found in [24–28], rely on measurements of spot speed from point detectors to estimate link travel time (LTT). Li et al. [24] compared the performance of four speed-based travel time estimation methods: an instantaneous model, a time slice model, a dynamic time slice model, and a linear model. By using a space-time grid method to reconstruct the vehicle trajectories, Van Lint and van der Zijpp [25] presented a new travel time estimation algorithm based on a linear function of speed. Cortes et al. [26] used an iterative scheme to calculate travel time and simulation results showed that their model had good estimation performance.
2. Cumulative plot based methods have been studied by [29–32]. A cumulative plot is a graph that describes the variation of cumulative traffic volumes over time on a certain segment of the road. By measuring the cumulative volumes upstream and downstream of a link, this method can be used to approximately estimate the link travel time. Bhaskar et al. [29] presented a travel time estimation method based on cumulative plots for urban signalized intersections. They also considered the impact of mid-link sources and sinks on the performance of travel time estimation via methods proposed in [30].
3. Regression models have been developed by [33–36]. In these models, link travel time is treated as the dependent variable and traffic information collected from point detectors (i.e. volume, occupancy, or speed) are considered as the independent variables By constructing the relationship between variables based on historical data, travel time can be calculated using regression models. Zhang and He [33] proposed regression models based on spot speed measurements from fixed points and the degree of saturation of corresponding links. Kwon et al. [34] formulated their regression model using flow, occupancy, time of departure, and day of week.
4. Artificial intelligence, in which neural network (NN) models have been widely used, has been studied in the context of travel time estimation by [37–40]. Naranjo et al. [37] designed a floating car data (FCD) augmentation system in which they used speed data obtained from loop detectors to estimate the speed of probe vehicles through neural networks. Liu et al. [38] presented a neural network based traffic flow model to estimate urban arterial travel time. The prediction performance from empirical data and simulation data indicated the accuracy and effectiveness of the neural network based traffic flow model.

The purpose of this work is to construct a high accuracy travel time estimation method based on loop detector data collected along a freeway. Although a large body of work has been published on the topic of travel time estimation, it is still difficult to estimate the travel time accurately through indirect methods such as those that use data collected from existing loop detectors. In this paper, we propose a new travel time estimation model based on an evolving fuzzy neural network using traffic flow data collected from existing loop detectors. Comparing with traditional methods, the EFNN based model in this study has better learning ability, which includes two parts: (1) the unsupervised learning process; In this process, define the fuzzy membership functions according to the clustering results. As the number of input samples increases, the cluster centers and membership functions are adjusted. The membership degree is determined by the distance between each data point in the input space and the new cluster centers. (2) the supervised learning process; Use the Takagi-Sugeno model to complete the fuzzy inference. The output errors (errors between the actual and estimated travel time) are used to adjust the inferring weights in the inference system. Furthermore, comparisons between the proposed method and some other travel time estimation methods such as an instantaneous model, a linear model, a cumulative plot based method, a multiple linear regression method, and an alternate neural network based approach are used to verify the effectiveness of the proposed method in scenarios using both actual and simulated data. We will henceforth refer to scenarios using actual data as actual scenarios and scenarios using simulated data as simulation scenarios.

## Data Description

The data sources in this study come from both actual and simulated scenarios. For the actual dataset, the loop detector data come from DRIVENET (Digital Roadway Interactive Visualization and Evaluation Network, http://uwdrive.net/STARLab), and travel time data come from HERE data source. HERE is a product of the communications in company Nokia. HERE can integrate a number of mapping and data collection services, including real-time data from GPS, smart phones, and consumer and commercial sources. The freeway travel time is derived from HERE traffic information. We selected three links (I5, I90 and I405) as testing examples. The links we selected for the case study serve high traffic volumes and experience high levels of congestion. In order to examine variability of travel time in different time periods, we selected three periods across the day including: morning peak (6:00–10:00), noon off-peak (11:00–14:00), and evening peak (16:00–20:00) to test performance of the proposed method.

The specific locations of loop detectors are marked along the links for both the actual and simulation scenarios. Although traffic flows in both directions along each link, we only consider travel time estimation for travel in one direction along each link: the north in I5, the east in I90 and the north in I405. Additionally, although there are two different lane types along the links, high occupancy vehicle (HOV) lanes and general purpose (GP) lanes, this study only focuses on the estimation of travel time for the GP lanes.

Travel times and traffic flow data (including volume, occupancy, and speed measurements) were collected at five-minute intervals for each link. Data used in the study were collected over a period of 15 days from September 10th to 25th, 2013. Data from the first 10 days were used to train and optimize the model, while data from the last five days were used to test the proposed method. More detailed information about the links is provided in Table 1.

**Table 1:** Segment information of three links.

Name	Upstream	Downstream	Length(km)	Number of main lanes
**I5N**	NE 175th Street	WA104	1.42	4
**I90E**	SE 34th Street	Lakemont Blvd SE	1.95	4
**I405N**	WA 520	NE 60th Street	2.83	4

**Fig 1:**
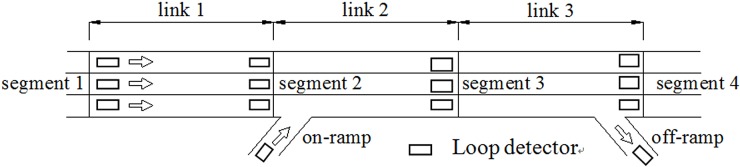
Simulation links with on and off ramp.

For the simulation dataset, we used Vissim to complete the simulation process. The traffic flow and travel time data were aggregated at one-minute intervals, and thus, 1,440 samples were recorded for each simulated day. Fig 1 shows a schematic of the study links with GP lanes. In order to consider the effect of ramps on travel time estimation for the mainline lanes, the on-ramp located upstream of link 2 and the off-ramp located downstream of link were regarded as a source and sink of data, respectively. The total simulation process lasted three days; samples from the first two days were used as the training dataset and the remainder of the data was used as the testing dataset. More information on the links can be found in Table 2.

**Table 2:** Segment information of links in simulation.

Name	Upstream	Downstream	Length(km)	Number of main lanes
**Link1**	Segment1	Segment 2	0.8	3
**Link2**	Segment 2+on-ramp	Segment 3	0.8	3
**Link3**	Segment 3	Segment 4+off-ramp	0.8	3

## Methodology

### Structure of the algorithm

The evolving fuzzy neural network model (EFNN) was presented in [41]. It has five layers. The first layer is the input layer, in which the input variables are stored and each node represent a variable. The second layer of nodes quantifies the fuzzy values of the input variables by transforming the input values into membership degrees to which they belong to the membership functions. Each node in the second layer represents a membership function. The number of membership functions and the formulation of each can change during the learning process. In the third layer, the rule nodes can evolve through supervised or unsupervised learning. For this layer, A denotes the activation of the rule nodes, and each rule node *r* is defined by two vectors of connection weights: W_1_(*r*) and W_2_(*r*). The former can be adjusted by unsupervised learning based on similarity measures, and the latter can be adjusted by supervised learning based on the output errors. Between the second and third layers, there is a short-term memory layer which connects to the rule layer and can be used to provide information to it via a feedback loop. The fourth layer of nodes represents fuzzy quantification of the output variables. Finally, the fifth layer represents the real values of the output variables. More details on the structure of an EFNN can be found in [41–43].

In this study, we combined the traffic flow data from loop detectors (volume, occupancy, and speed) and link travel times into a dataset that was processed using a FNN-based system capable of learning traffic behavior. In this system, traffic flow data were the inputs and link travel time was the output. Once the system had been trained, it was able to use the real-time traffic flow data to estimate travel time. We adopted an improved EFNN in this study. Compared to a traditional FNN, an EFNN makes use of improved learning processes, which include two parts: the unsupervised learning process and the supervised learning process. In the unsupervised learning process, the main purpose is to determine parameters in fuzzy variables’ membership functions. The supervised learning process is then used to adjust weights in the fuzzy inference system.

### Clustering of traffic flow based on K-means method

The aim of the K-means method is to classify the *l* input samples which take the form of *m*-vectors *x*_i_ = {*x*_i1_, *x*_i2_, …, *x*_il_}, *i* = 1,2, …, *m*, (here *m* = 6, *l* is the number of samples) into *n* clusters, and determine the cluster centers for each cluster under the condition of minimizing an objective function *J*. The distance between *x*_i_ and the cluster center *c*_j_ is first defined in the following equation:

$$d(x_{i},c_{j})=\sum_{k=1}^{l}\left|x_{ik}-c_{j}\right|$$ (1)

where, |·| represents the general Euclidean distance. Then the objective function is defined as:

$$J=\sum_{i=1}^{m}\sum_{j=1}^{n}d(x_{i},c_{j})$$ (2)

The algorithm for determining the cluster centers with the K-means clustering method can be divided into three processes. First, initialize the cluster center *c*_j._ Second, iteratively modify the partition to reduce the sum of the distances for each sample from the centers of the cluster to which the samples belongs. Finally, the process terminates if one of following conditions is satisfied: the value of objective function is below a certain tolerance; the difference in the values of the objective function between adjacent iterations is less than a preset threshold; or the iteration process is complete.

**Fig 2:**
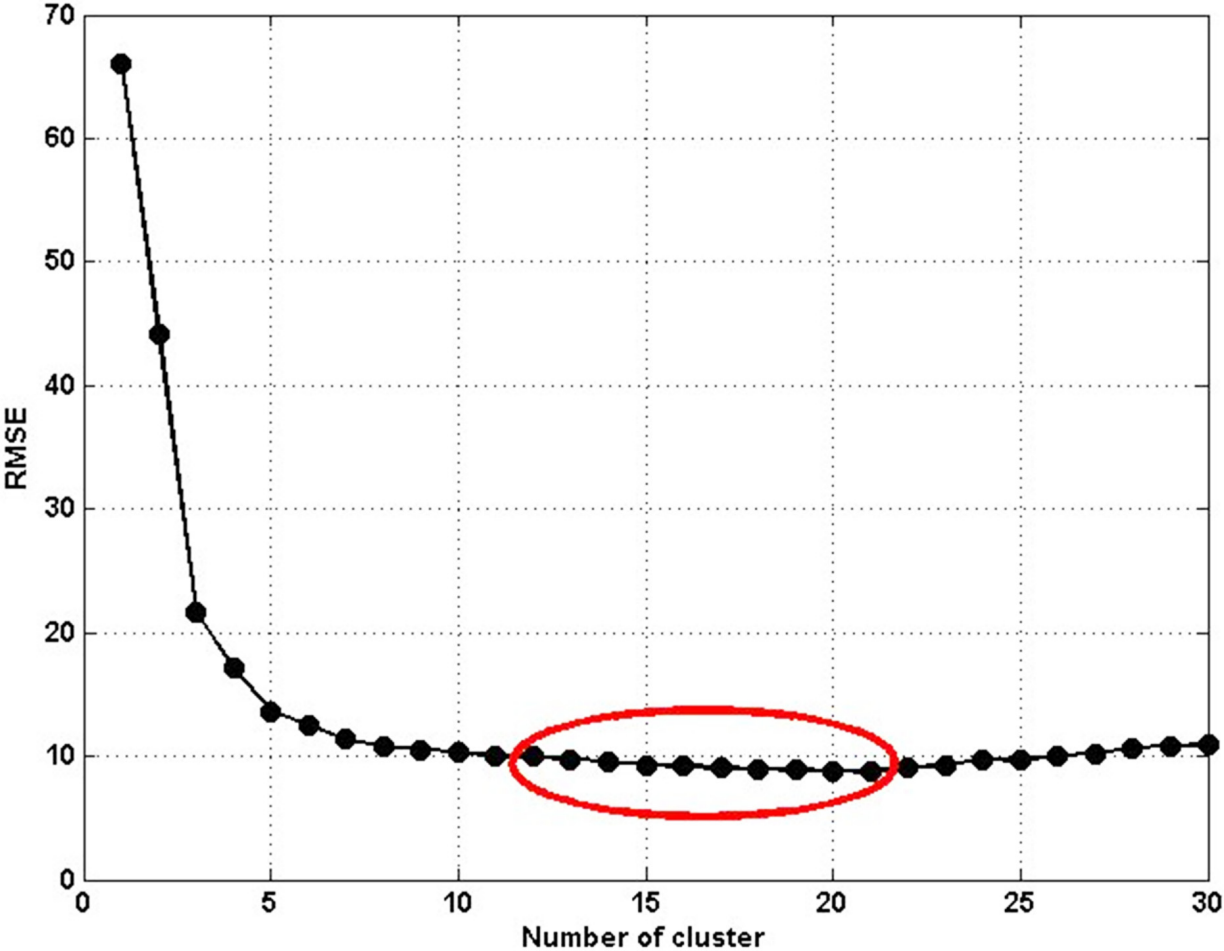
Selection of clusters number.

Here, we used data collected in I5 to analyze the influence of the cluster number to estimation results. Fig 2 shows the distribution of estimation errors with different number of clusters. We use RMSE as the evaluating index, the number of cluster *n* increases from 1 to 30. As we can see, when value of *n* is small (*n*<5), the estimation error is high. The reason is that K-means method is unable to effectively divide the traffic flow into different patterns with small cluster number, which also leads to poor learning effect. When value of n is large (*n*>22), the estimation error rises slightly. The reason is that K-means method would classify the traffic flow data into sparse patterns with large *n*, which can destroy the association in raw data. Furthermore, large number of clusters will result in the increase of parameters’ number in the training process, such as the number of fuzzy rules and fuzzy membership function, see Eq (4). Finally, a large amount of parameters in Takagi-Sugeno inference would cause over fitting in training process to a certain extent. When the value of *n* is in the range of 6 to 21, the estimation errors decrease and become stable. We also find the similar trends for the data in I90 and I405. Although the RMSE reaches its minimum value at *n* = 20, the value of *n* is suggested be selected in the range of 2*m to 3.5*m (*m* is the dimension of input samples, here, *m* = 6), for which the estimation accuracy is high and stable.

**Fig 3:**
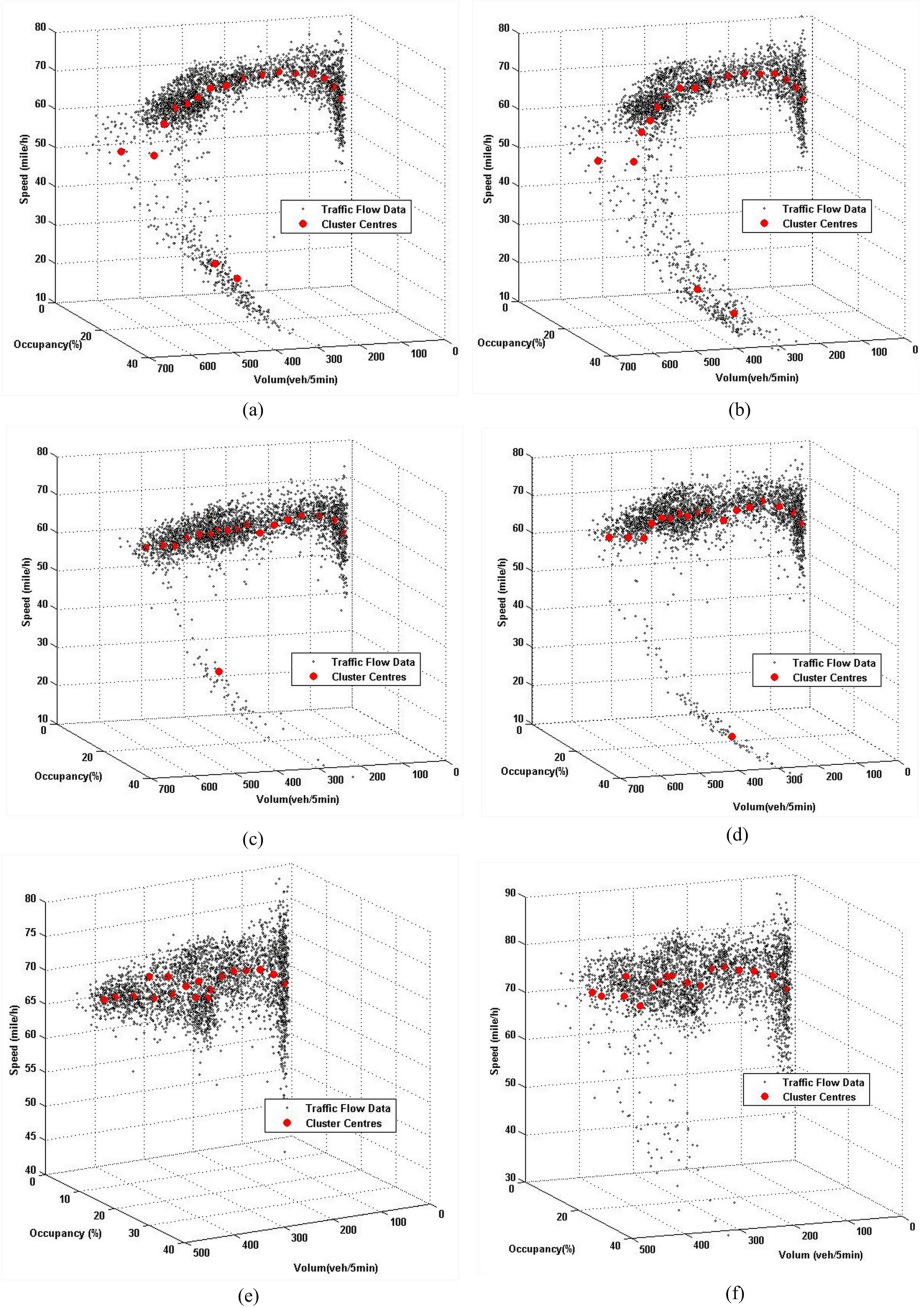
Clustering results of observed traffic flow data by K-means method. (a) Upstream of I5. (b) Downstream of I5. (c) Upstream of I90. (d) Downstream of I90. (e) Upstream of I405. (f) Downstream of I405.

**Fig 4:**
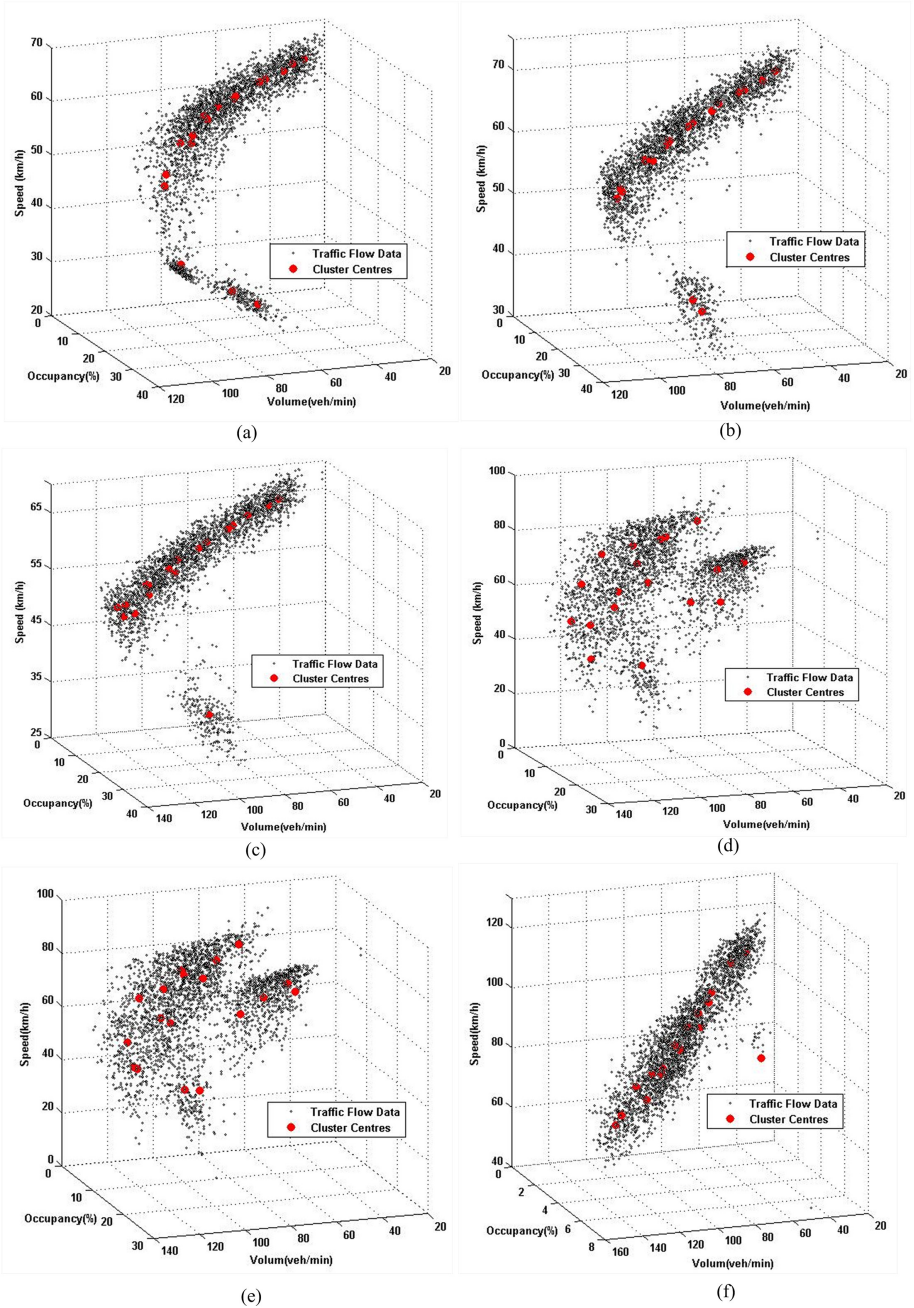
Clustering results of simulating traffic flow data by K-means method. (a) Upstream of Link1. (b) Downstream of Link1. (c) Upstream of Link2. (d) Downstream of Link2. (e) Upstream of Link3. (f) Downstream of Link3.

Ultimately, the K-means method was used to partition the traffic flow data from the actual and simulation scenarios into 18 clusters; the results of the clustering can be seen in Figs 3 and 4. In each of the aforementioned figures, the black points represent the data samples and red points show the cluster centers.

### Travel time estimation based on EFNN

In the EFNN, we used a Takagi-Sugeno type fuzzy inference system to construct fuzzy rules. As each sample, *x* = [*x*_1_,*x*_2_,…,*x*_*m*_], has *n* memberships describing the degree to which it belongs to each cluster, the number of rules is equal to the number of clusters *n*. The rules are shown as follows:

if *x*_1_ is *R*_11_ and *x*_2_ is *R*_12_ and … and *x*_m_ is *R*_1m_, then *y* is *f*_1_(*x*_1_, *x*_2_, …, *x*_m_)
if *x*_1_ is *R*_21_ and *x*_2_ is *R*_22_ and … and *x*_m_ is *R*_2m_, then *y* is *f*_2_(*x*_1_, *x*_2_, …, *x*_m_)
… …
if *x*_1_ is *R*_n1_ and *x*_2_ is *R*_n2_ and … and *x*_m_ is *R*_nm_, then *y* is *f*_n_(*x*_1_, *x*_2_, …, *x*_m_)

where *R*_ij_ indicates a fuzzy set defined by its membership function, *x*_j_ is the antecedent variable, and *f*_i_ is the inference consequence of variable *y* when the *i*th rule is employed. In this study, the fuzzy membership functions were selected to be of the Gaussian type with two parameters defined as follows:

$$mf(x)=e^{-\frac{{\left(x-\mu \right)}^{2}}{\sigma^{2}}}$$ (3)

where, *mf* is defined as membership function, *μ* is the value of the cluster center on the *x* dimension, *σ*^2^ is the variance of the distance between input samples and the cluster center on the *x* dimension. Overall, the total number of membership functions is *n*×*m*. In the model, we used a first-order Takagi-Sugeno type fuzzy inference system which means the function *f*_*i*_(*x*_1_,*x*_2_,…,*x*_*m*_), i = 1,2,…*n*, is a linear function. So, for an input data point $x^{0}=[x_{1}^{0},x_{2}^{0},\ldots ,x_{m}^{0}]$, the inferring results of the system, *y*^0^, can be calculated as the weighted average of outputs from each rule:

$$y^{0}=\frac{\sum_{i=1}^{n}w_{i}\cdot f_{i}(x_{1}^{0},x_{2}^{0},\ldots ,x_{m}^{0})}{\sum_{i=1}^{n}w_{i}}$$ (4)

where, $w_{i}=\prod_{j=1}^{m}mf_{R_{ij}}(x_{j}^{0})$; *i* = 1,2,…,*n*, *j* = 1,2,…,*m*.

In the learning process, we used a least squares estimator (LSE) [44–45] to train the linear functions. Each of the linear function can be described as follows:

$$y=\alpha_{0}+\alpha_{1}x_{1}+\alpha_{2}x_{2}+\ldots +\alpha_{m}x_{m}$$ (5)

The training dataset included *p* data pairs, {([*x*_*i*1_,*x*_*i*2_,…*x*_*im*_], *y*_i_), *i* = 1,2,…,*p*}, and was used to calculate the coefficients *a* = [*a*_0_*a*_1_*a*_2_…*a*_*m*_]^T^via the following equation based on LSE:

$$a={({\text{A}}^{\text{T}}\text{A)}}^{\text{-1}}{\text{A}}^{\text{T}}y$$ (6)

where

$$\text{A}=\left(\begin{array}{ccccc} 1 & x_{11} & x_{12} & \cdots & x_{1m}\\ 1 & x_{21} & x_{22} & \cdots & x_{2m}\\ \vdots & \vdots & \vdots & \vdots & \vdots \\ 1 & x_{p1} & x_{p2} & \cdots & x_{pm} \end{array}\right)$$

and

$$y={\left[y_{1}y_{2}y_{3}\ldots y_{p}\right]}^{\text{T}}$$

Furthermore, we used an improving weighted least squares estimation method in [44–45] to optimize the parameters.

$$a_{w}={({\text{A}}^{\text{T}}\text{WA)}}^{\text{-1}}{\text{A}}^{\text{T}}\text{W}y$$ (7)

where

$$\text{W}=\left(\begin{array}{cccc} w_{1} & 0 & \cdots & 0\\ 0 & w_{2} & \cdots & 0\\ \vdots & \vdots & \vdots & \vdots \\ 0 & \cdots & \cdots & w_{p} \end{array}\right)$$

*w*_j_ represents the distance between the *j*th sample and the corresponding cluster center, *j* = 1,2,…,*p*. Eq (7) can be rewritten according to the following:

$$\{\begin{array}{c} {\text{P}}_{w}={\left({\text{A}}^{\text{T}}\text{WA}\right)}^{-1}\\ a_{w}={\text{P}}_{w}{\text{A}}^{\text{T}}\text{W}y \end{array}$$ (8)

**Fig 5:**
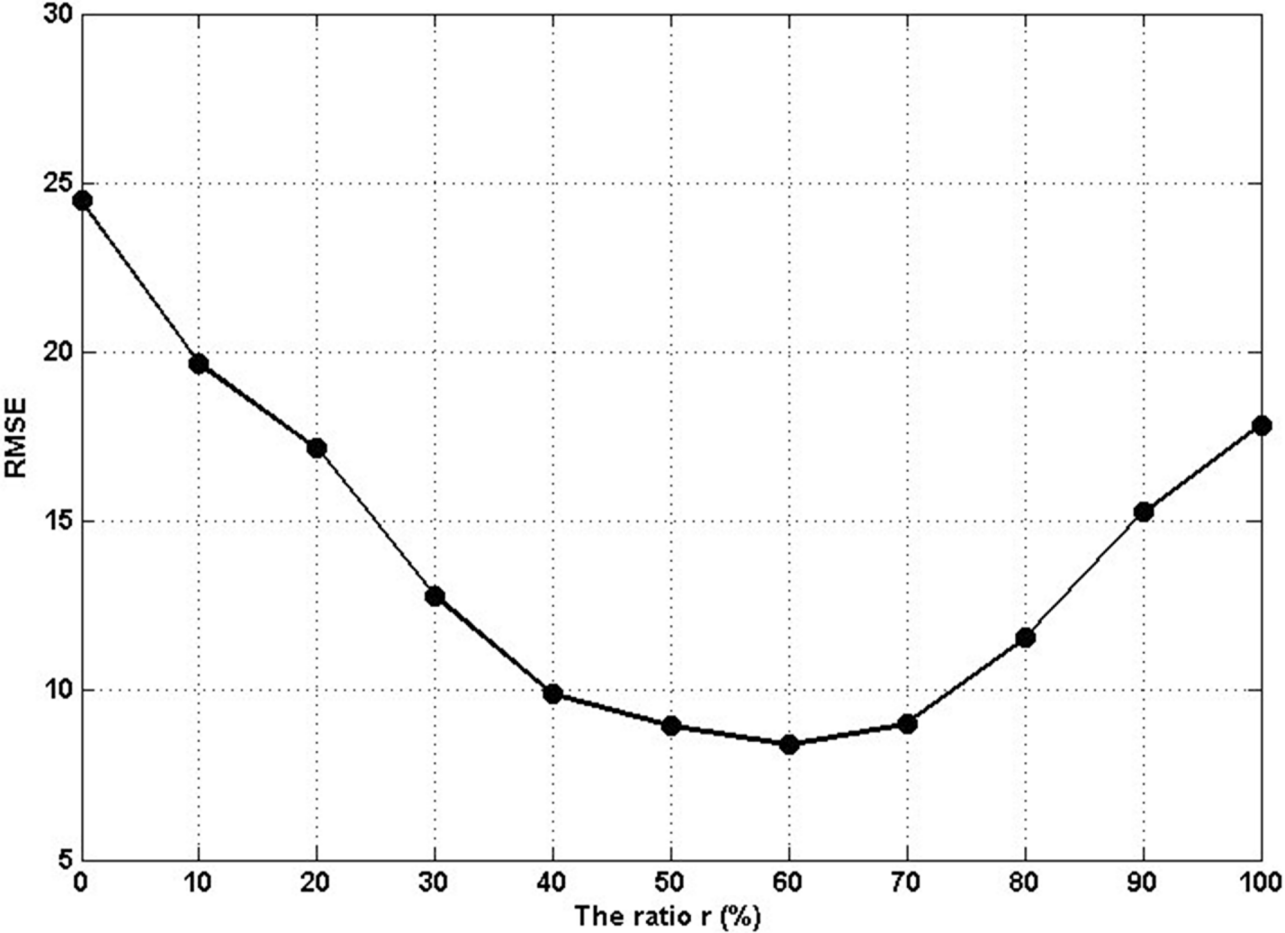
The influence of ratio *r* to estimation accuracy.

Define the *k*th row vector of matrix A in Eq (6) to be *b*^T^_k_ = [1 *x*_k1_ *x*_k2_ … *x*_km_] and denote the *k*th element of *y* as *y*_k_. Then the vector of coefficient *a* can be iteratively calculated by Eq (9) shown in the following. The calculation process uses a recursive, improved weighted LSE method in [43–45] to complete the optimization.

$$\{\begin{array}{c} a_{k+1}=a_{k}+w_{k+1}{\text{P}}_{k+1}b_{k+1}(y_{k+1}-b_{k+1}^{T}a_{k})\\ {\text{P}}_{k+1}=\frac{1}{\lambda }({\text{P}}_{k}-\frac{w_{k+1}{\text{P}}_{k}b_{k+1}b_{k+1}^{T}{\text{P}}_{k}}{\lambda +b_{k+1}^{T}{\text{P}}_{k}b_{k+1}^{}}) \end{array}\;k=t,\;t+1,\ldots ,p-1$$ (9)

where λ is the forgetting factor and with a value is generally between 0.8–1.0, *a*_t_ and *P*_t_ are the initial values of *a* and *P*, which can be calculated in Eq (8) by using the first *t* data pairs from the training dataset. Here, we discussed the influence of sample splits to the estimation performance. We define the *r* as the split ratio, if *p* represents the total number of training samples, then r*p is the number of samples used in first step and (1-*r*)*p indicates the number of samples used in second step. The Fig 5 displays the relationship between the ratio *r* and estimation errors RMSE in link of I5. When the value of *r* is too small (*r*<0.4) or too large (*r*>0.7), the RMSE is high. When the value of *r* ranges from 0.4 to 0.7, the RMSE is relative low and stable. The reason is that the small value of *r* will result in improper initial parameters from Eq (8), then these initial values are used to update parameters in Eq (9), finally the errors will be cumulated and cause inaccurate travel time estimation. Similarly, the large value of *r* will result in the final parameters excessively depend on initial values, and error adjustment mechanism plays only a small role in learning process. In summary, we set the value of *r* to 0.5 in this study, which can not only guarantee proper initial parameters but also make full use of the function of error adjustment.

Thus, the *t* equals to 0.5*p. The remaining (*p*-*t*) data pairs are used to iteratively optimize the values of coefficients.

## Experiment and Results Analysis

### Measurements of effectiveness

Three common indicators are used to evaluate and compare different estimation methods: mean absolute error (MAE), root mean square error (RMSE), and mean absolute relative error (MARE). The three criteria are defined in the following equations:

$$\text{MAE}=\frac{1}{n}\sum_{i=1}^{n}\left|t_{i}-{\hat{t}}_{i}\right|$$ (10)

$$\text{RMSE }=\;\sqrt{\frac{1}{n}\sum_{i=1}^{n}{\left(t_{i}-{\hat{t}}_{i}\right)}^{2}}$$ (11)

$$\text{MARE(\%)}=\frac{1}{n}\sum_{i=1}^{n}\frac{\left|t_{i}-{\hat{t}}_{i}\right|}{t_{i}}\times 100$$ (12)

where, *n* is the number of testing samples, *t*_i_ denotes the observed values of travel time in the actual or simulation scenarios, and $\hat{t}$_i_ indicates the values of travel time that are the outputs of the estimation methods.

### Results comparison for actual scenario

In the model validation, we compare the travel time estimation performance of the EFNN with other commonly used methods: multiple linear regression (MLR), instantaneous models (IM) [24], linear models (LM) [25], neural networks (NN) [37] and cumulative plots (CP) [29] method.

For the MLR, the calculation process can be expressed as:

$$y(k)=\alpha_{0}+\sum_{i=1}^{n}\alpha_{i}\cdot x_{i}(k)$$ (13)

where *x*_i_ is the *i*th independent variable, *n* is six (here, we consider volume, occupancy, and speed at upstream and downstream of a given link as independent variables), *y* indicates the dependent variable: link travel time, *α*_0_ and *α*_i_ are the regression coefficient, *k* is the time index.

For the IM, it can be shown as:

$$t(k)=\frac{2l}{v_{a}(k)+v_{b}(k)}$$ (14)

where *v*_a_(*k*) and *v*_b_(*k*) denote the average speeds at upstream and downstream of a link at time *k*, *l* is the length of a given link, *t*(*k*) represents the link travel time.

For the LM,

$$v(k)=v_{a}(k)+\frac{x(k)-x_{a}}{x_{d}-x_{a}}(v_{d}(k)-v_{a}(k))$$ (15)

where *x*_a_ and *x*_b_ are the locations of upstream and downstream segment for a given link, *x*(*k*) is the location of a vehicle at time *k*, *v*_a_(*k*) and *v*_b_(*k*) denote the average speeds at upstream and downstream of a link at time *k*, *v*(*k*) represents the speed of vehicle at location *x*(*k*). According to the values of *v*(*k*), the trajectory for a given vehicle can be reconstructed. Furthermore, the link travel time can be calculated with the trajectories in space-time plots.

For the NN, the input variables are the volume, occupancy, and speed at upstream and downstream of a given link, and the output variable is the link travel time. For the model structure, we use one hidden layer and 50 neurons for the application in actual and simulation scenarios.

For the CP method, the link travel time can be estimated from the cumulative volume of upstream and downstream in a given link.

For the EFNN method, input variables are the volume, occupancy, and speed at upstream and downstream of a given link, and the output variable is the link travel time. Before the model application, we should determine optimal cluster number, number of fuzzy rules and split ratio. Travel time estimation performance results for each of the different methods aforementioned, when applied to the three real-world links are shown in Tables 3, 4 and 5, respectively. From observation of the tables, we can see that the EFNN method obtained better estimation performance than the other four methods. As a consequence of only considering the speed at points upstream and downstream of a given segment, travel times estimated from the IM are easily affected by the variation of speed. Thus, the IM method often overestimated or underestimated the actual travel time values. The LM method is considered an improvement of the IM method. It calculates the average speed between points upstream and downstream of a given section and estimates travel time based on reconstructed trajectories. Although the LM method obviously enhanced the estimation accuracy compared to the IM method, linear models can have difficulty in accurately describing the variation of speed along a link based on the underlying linear assumption. Furthermore, estimation performance of LM is also easily influenced by speeds at the extremities of the links. The EFNN method obtained better estimation performance than the IM and LM methods due to its learning ability. In the EFNN, the volume, occupancy, and speed data collected from upstream and downstream detectors are classified into different clusters to reflect variation of traffic flow patterns. In the training process, the traffic flow data in different patterns are considered as input variables and corresponding travel time as the output variable. With the aim to best describe different patterns, parameters are optimized to accurately describe the non-linear relationship between traffic flow and travel time. Link travel time can then be estimated from the trained model based on real-time, multisource traffic flow data.

**Table 3:** Comparison of estimating results for link in I5.

Time of day		Estimation Methods				
	MOE	EFNN(N = 20)	MLR	IM	LM	NN
**Morning6:00–10:00**	MAE	7.23	12.33	16.76	13.53	10.78
	RMSE	11.73	16.54	19.14	15.67	14.43
	MARE (%)	5.27	9.86	12.35	11.45	7.75
**Noon11:00–14:00**	MAE	3.32	5.42	10.02	8.98	4.72
	RMSE	5.11	8.53	12.52	11.25	6.24
	MARE (%)	2.85	5.93	9.02	7.58	3.30
**Evening16:00–20:00**	MAE	7.45	12.35	20.72	15.14	11.83
	RMSE	11.36	18.52	22.99	20.58	17.38
	MARE (%)	6.69	10.47	14.62	12.92	9.87
**All day**	MAE	4.25	8.09	15.46	12.02	6.97
	RMSE	7.94	15.71	18.51	14.9	11.51
	MARE (%)	3.81	7.72	11.12	9.67	6.06

**Table 4:** Comparison of estimating results for link in I90.

Time of day		Estimation Methods				
	MOE	EFNN(N = 18)	MLR	IM	LM	NN
**Morning6:00–10:00**	MAE	6.53	7.77	9.92	8.05	6.94
	RMSE	8.72	10.71	15.43	13.55	11.15
	MARE (%)	2.78	3.94	7.75	5.01	4.28
**Noon11:00–14:00**	MAE	3.17	3.59	4.36	4.04	3.94
	RMSE	4.74	5.15	5.89	5.47	4.65
	MARE (%)	1.35	1.43	1.74	1.46	1.32
**Evening16:00–20:00**	MAE	3.15	8.61	6.05	5.93	3.49
	RMSE	3.62	10.87	9.54	9.37	4.98
	MARE (%)	1.09	3.67	2.98	2.74	1.75
**All day**	MAE	3.15	6.45	6.91	6.27	4.82
	RMSE	4.21	9.71	10.95	9.42	6.45
	MARE (%)	1.63	3.28	3.67	2.81	2.25

**Table 5:** Comparison of estimating results for link in I405.

Time of day		Estimation Methods				
	MOE	EFNN(N = 20)	MLR	IM	LM	NN
**Morning6:00–10:00**	MAE	12.35	16.08	19.92	16.60	13.69
	RMSE	18.88	21.68	25.08	21.84	21.17
	MARE (%)	8.33	12.08	14.74	12.25	9.34
**Noon11:00–14:00**	MAE	3.23	8.41	8.99	6.41	4.78
	RMSE	4.36	9.05	9.15	7.05	6.67
	MARE (%)	1.09	2.40	2.27	1.74	1.36
**Evening16:00–20:00**	MAE	3.32	6.98	3.47	3.37	3.10
	RMSE	3.96	8.25	4.62	4.15	3.76
	MARE (%)	0.97	2.09	1.34	1.03	0.92
**All day**	MAE	6.05	10.56	11.17	10.17	8.86
	RMSE	11.65	17.18	16.14	15.59	15.21
	MARE (%)	1.67	3.24	3.01	2.89	2.77

To consider different patterns of travel time according to the time of day, we also compared the results in three periods for the five days of data that comprised the test set: morning, noon, and evening. Volumetric peaks are common during the morning and evening periods on the facilities considered in this study, and travel times in these periods exhibit obvious fluctuations. During the noon period, however, the travel times become comparatively stable. From Tables 3, 4 and 5, we can see that the estimation errors for all the methods in morning and evening periods were generally higher than those for the noon period.

**Fig 6:**
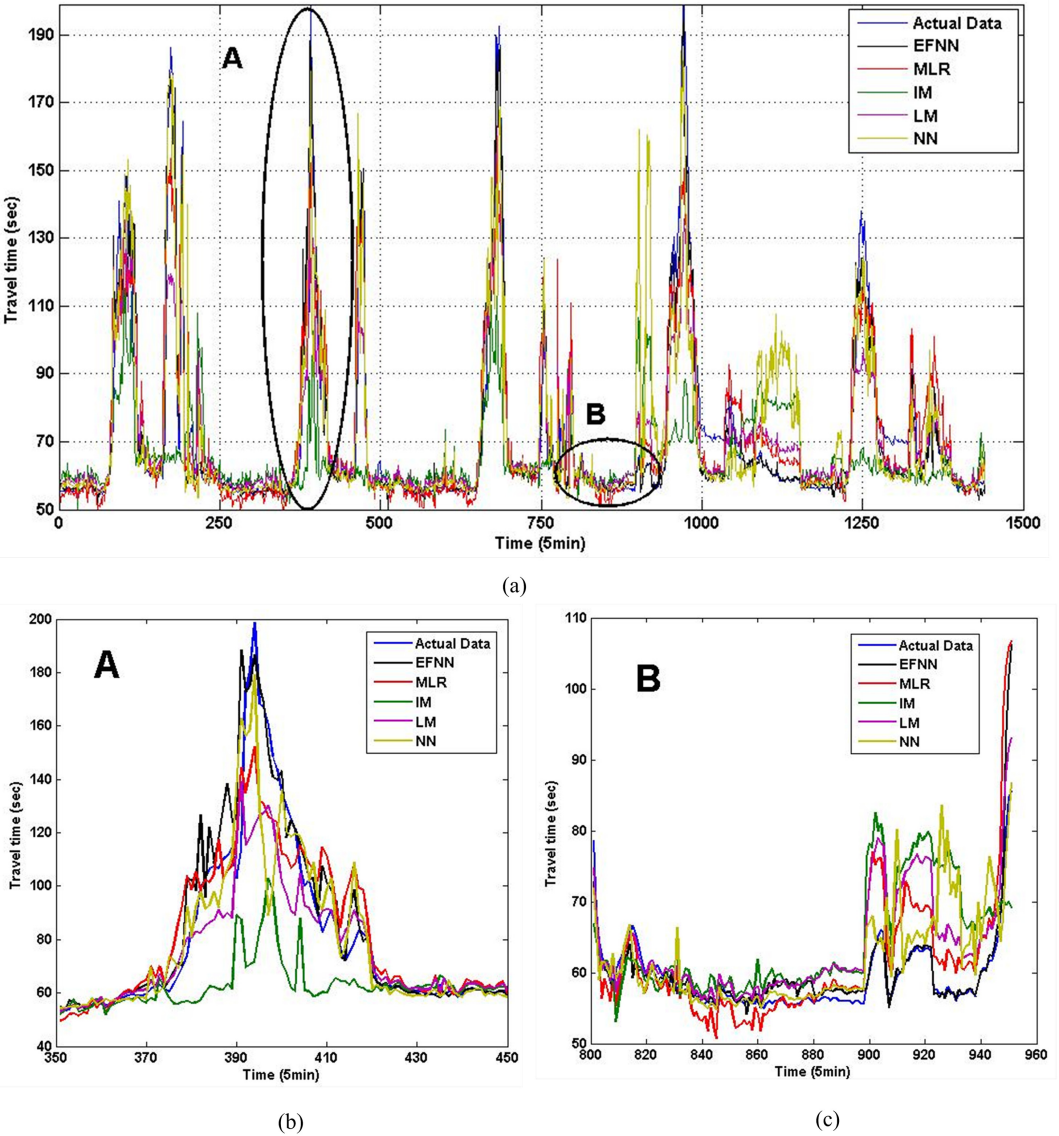
The estimated travel time in three periods for link in I5. (a) Estimation comparison in five days. (b) Peak hours. (c) Non-peak hours.

**Fig 7:**
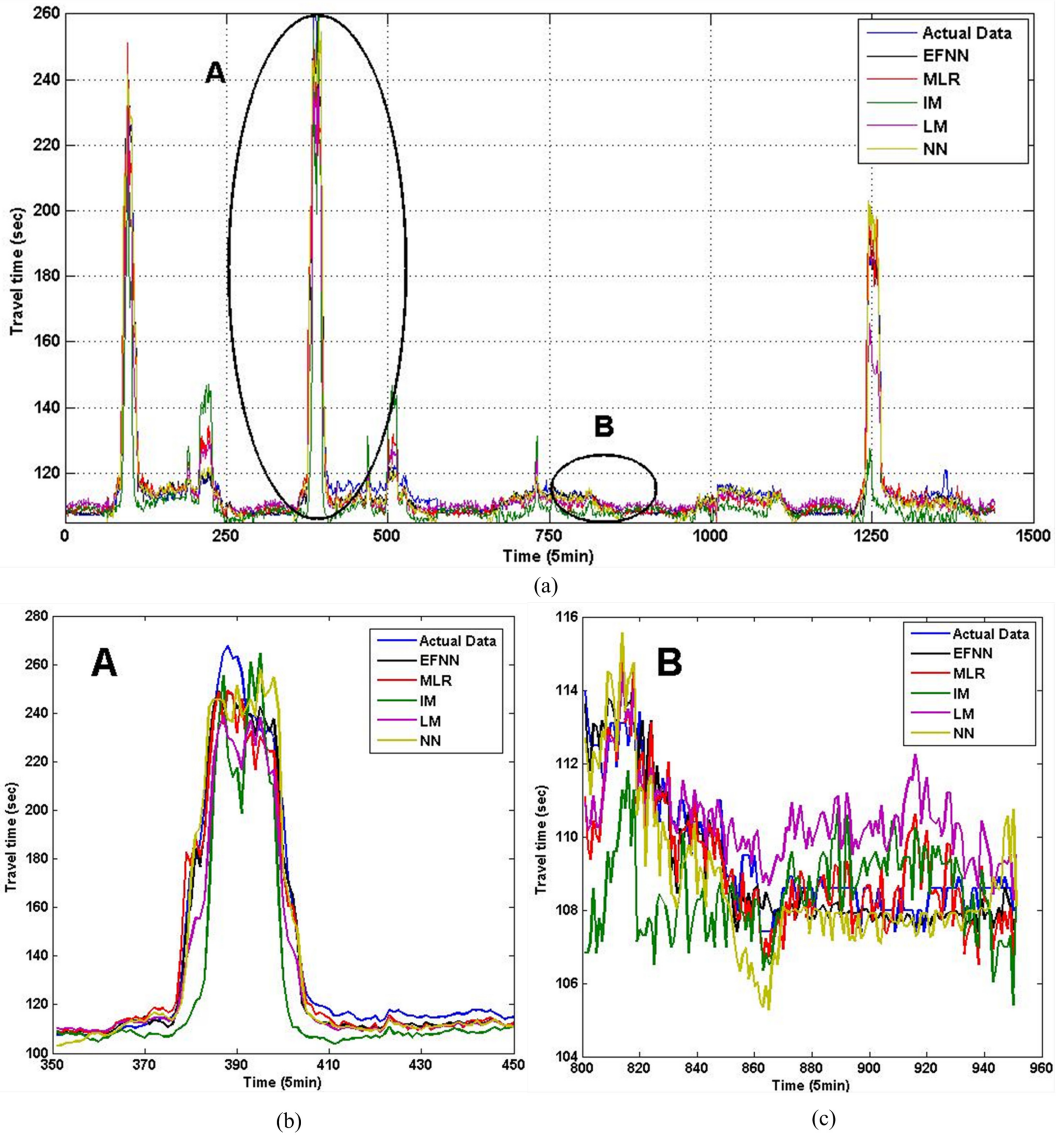
The estimated travel time in three periods for link in I90. (a) Estimation comparison in five days. (b) Peak hours. (c) Non-peak hours.

**Fig 8:**
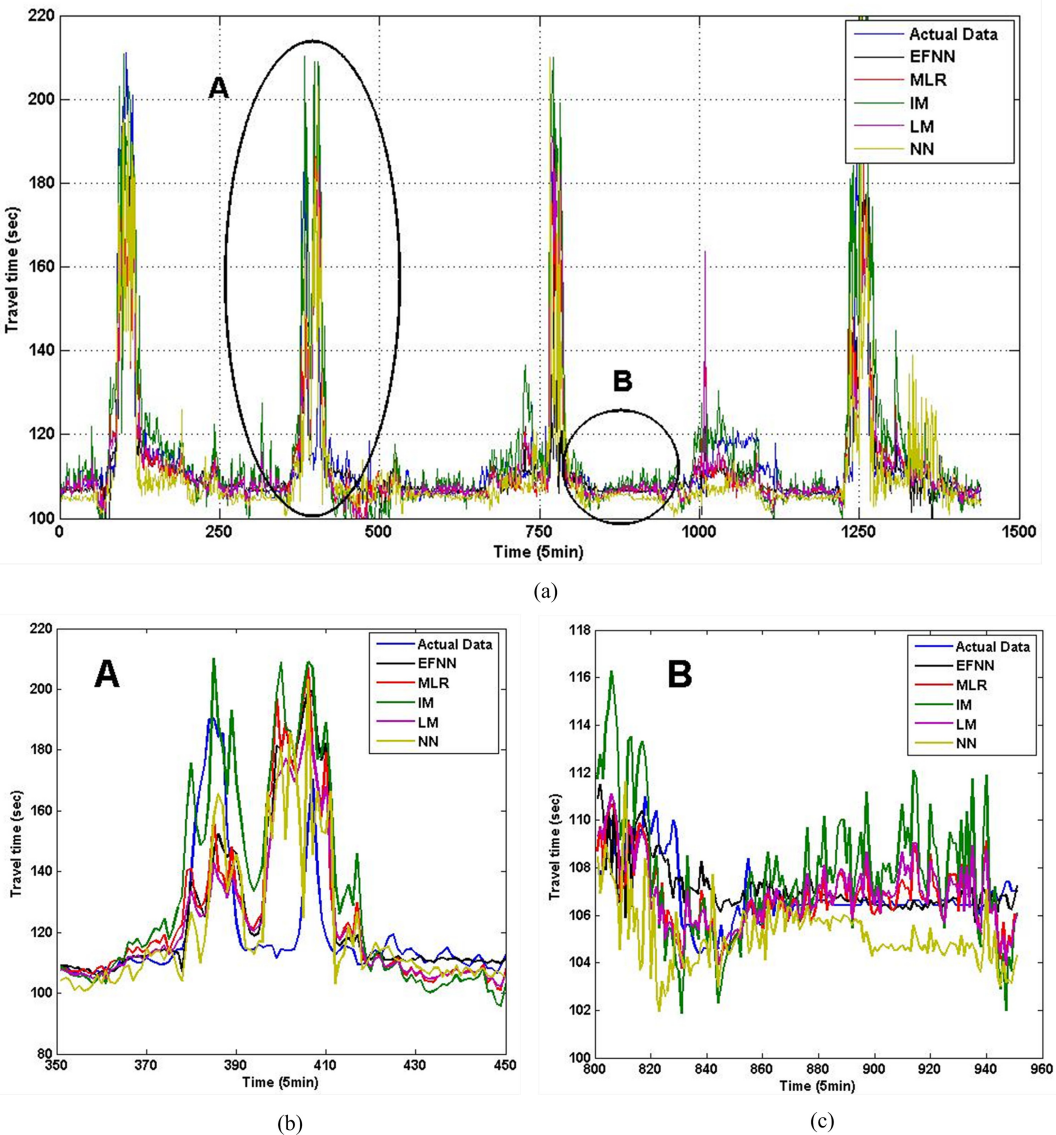
The estimated travel time in three periods for link in I405. (a) Estimation comparison in five days. (b) Peak hours. (c) Non-peak hours.

Fig 6a provides the estimation results of I5 in five days. Fig 6b and 6c are estimation comparison in peak hours and non-peak hours. As we have mentioned previously, travel time under congested conditions generally varies intensively, and it tends to be more stable in non-peak hours. Thus, compared to estimation errors in a non-peak period, the estimation errors under congested conditions were much higher. Furthermore, we compare estimation performance of methods in three time periods in Table 3. For I90 and I405, we obtain similar analyzing results, which can be seen in Figs 7, 8 and Tables 4, 5. The *N* is tables means number of fuzzy rules in EFNN.

### Results comparison for simulation scenario

In the simulation, the input and output variables for the EFNN, MLR, and NN methods were the same as for the models used to predict travel time in the actual scenario. The cumulative plots (CP) method is another traditional approach used to estimate link travel time. In applications using actual data, due to various reasons, the traffic volume data collected from loop detectors are liable to be missing or inaccurate. These issues could significantly influence the estimation accuracy of the cumulative plots method. In simulation, the volume data can be accurately collected, so we can compare the CP method with the other estimation methods in the simulation.

In the Tables 6, 7 and 8, we compared the travel time estimation results of the proposed model with other six methods for the three links. The *N* is tables means number of fuzzy rules in EFNN. Since it only uses cumulative volume to approximately estimate the link travel time, the CP method cannot acquire high estimation accuracy. For all the testing samples, the EFNN and NN methods were superior to the other four methods. The CP method slightly outperformed the IM method, and the LM method consistently showed improved estimation ability over the IM method. In the simulation, we also examined the estimation performance of all the methods in three time periods in terms of MAE, RMSE, and MARE. The samples sizes of the testing datasets used for three periods were also 240, 180, and 240, as were used for the actual scenario. From comparing the results from the simulations, we observed that the estimation errors for all of the methods in evening periods were generally higher than those for the morning and the noon periods as there was a peak in the evening periods.

**Table 6:** Comparison of estimating results for Link 1.

Time of day		Estimation Methods					
	MOE	EFNN(N = 16)	MLR	IM	LM	NN	CP
**Morning6:00–10:00**	MAE	1.18	2.35	5.06	2.14	1.66	4.70
	RMSE	1.78	3.17	7.70	3.09	2.45	5.90
	MARE (%)	2.43	4.34	9.16	3.92	3.05	9.13
**Noon11:00–14:00**	MAE	0.77	1.81	2.08	1.07	0.93	4.40
	RMSE	1.14	2.56	2.91	1.50	1.50	5.47
	MARE (%)	1.58	3.76	4.29	2.18	1.90	9.16
**Evening16:00–20:00**	MAE	6.63	10.18	16.62	23.07	7.39	10.19
	RMSE	10.41	14.35	21.04	28.38	11.22	13.54
	MARE (%)	6.12	9.54	15.40	20.13	6.71	10.33
**All day**	MAE	1.87	3.57	5.62	6.02	2.46	6.42
	RMSE	4.73	7.10	10.58	13.28	5.47	8.74
	MARE (%)	2.56	4.74	7.26	6.36	3.10	11.02

**Table 7:** Comparison of estimating results for Link 2.

Time of day		Estimation Methods					
	MOE	EFNN(N = 18)	MLR	IM	LM	NN	CP
**Morning6:00–10:00**	MAE	0.93	1.71	12.22	1.41	1.12	4.22
	RMSE	1.34	2.19	12.66	1.87	1.54	5.39
	MARE (%)	2.33	4.27	31.51	3.46	2.78	10.89
**Noon11:00–14:00**	MAE	1.06	1.74	10.86	1.18	1.14	5.01
	RMSE	1.73	2.66	11.25	2.26	1.82	6.35
	MARE (%)	2.62	4.30	28.79	2.83	2.85	12.99
**Evening16:00–20:00**	MAE	3.77	5.76	12.72	7.06	4.46	10.26
	RMSE	5.54	7.74	14.26	10.05	6.16	12.69
	MARE (%)	6.15	8.71	22.19	9.92	6.61	16.57
**All day**	MAE	1.25	2.64	10.53	3.18	1.81	6.13
	RMSE	2.49	4.29	11.34	5.28	3.22	8.14
	MARE (%)	3.01	5.35	25.75	6.41	3.54	14.06

**Table 8:** Comparison of estimating results for Link 3.

Time of day		Estimation Methods					
	MOE	EFNN(N = 18)	MLR	IM	LM	NN	CP
**Morning6:00–10:00**	MAE	2.03	2.51	5.26	5.71	2.19	6.14
	RMSE	2.66	3.17	6.75	7.14	2.82	7.58
	MARE (%)	4.80	6.15	12.57	13.22	5.32	15.66
**Noon11:00–14:00**	MAE	1.94	2.19	4.74	5.01	2.08	6.27
	RMSE	2.78	3.01	6.14	6.35	2.86	7.73
	MARE (%)	4.83	5.54	11.86	12.25	5.25	16.74
**Evening16:00–20:00**	MAE	2.16	2.70	15.31	3.76	2.18	5.12
	RMSE	2.77	3.31	17.52	5.09	2.83	6.46
	MARE (%)	5.15	6.59	36.73	8.52	5.21	12.35
**All day**	MAE	1.89	2.24	9.07	4.35	2.08	6.21
	RMSE	2.56	3.01	11.46	5.63	2.74	7.71
	MARE (%)	4.90	5.95	25.25	11.24	5.58	17.48

In addition to evaluate estimation results using MAE, RMSE and MARE, we also conducted the Friedman test [46] to compare the proposed model with five methods. For the three considered datasets, the Friedman test results consistently suggest that the difference in travel time estimation error between the EFNN model and other four considered models is statistically significant.

### Results comparison with other fuzzy regression methods

We compare the estimation performance with two widely used fuzzy regression methods: Fuzzy Neural Network (FNN) [47] and Fuzzy C-means (FCM) [48,49]. In the FNN we used same Takagi-Sugeno inferring model as EFNN. In the FNN, we should determine the number of fuzzy rules. In the FCM, the number of cluster should be determined before application. In order to make fair comparison, we used same number of fuzzy rules in FNN and same number of clusters in FCM as EFNN (in EFNN, the number of fuzzy rules is equal to the number of clusters). The *N* indicates the number of clusters or number of fuzzy rules. We used same training and testing dataset to calibrate and validate models. From the estimation results in Table 9, we can see the EFNN obviously outperforms the FNN and FCM for its unsupervised and supervised learning process.

**Table 9:** Comparison of estimating results with other fuzzy regression methods.

Links		Estimation Methods		
	MOE	EFNN	FNN	FCM
**I5(N = 20)**	MAE	4.25	7.65	6.83
	RMSE	7.94	10.92	10.15
	MARE (%)	3.81	5.79	5.04
**I90(N = 18)**	MAE	3.15	6.04	4.93
	RMSE	4.21	9.55	7.05
	MARE (%)	1.63	2.87	2.19
**I405(N = 20)**	MAE	6.05	10.05	8.78
	RMSE	11.65	15.22	14.71
	MARE (%)	1.67	2.91	2.46

### The influences of link length and ramp to the estimation performance

**Fig 9:**
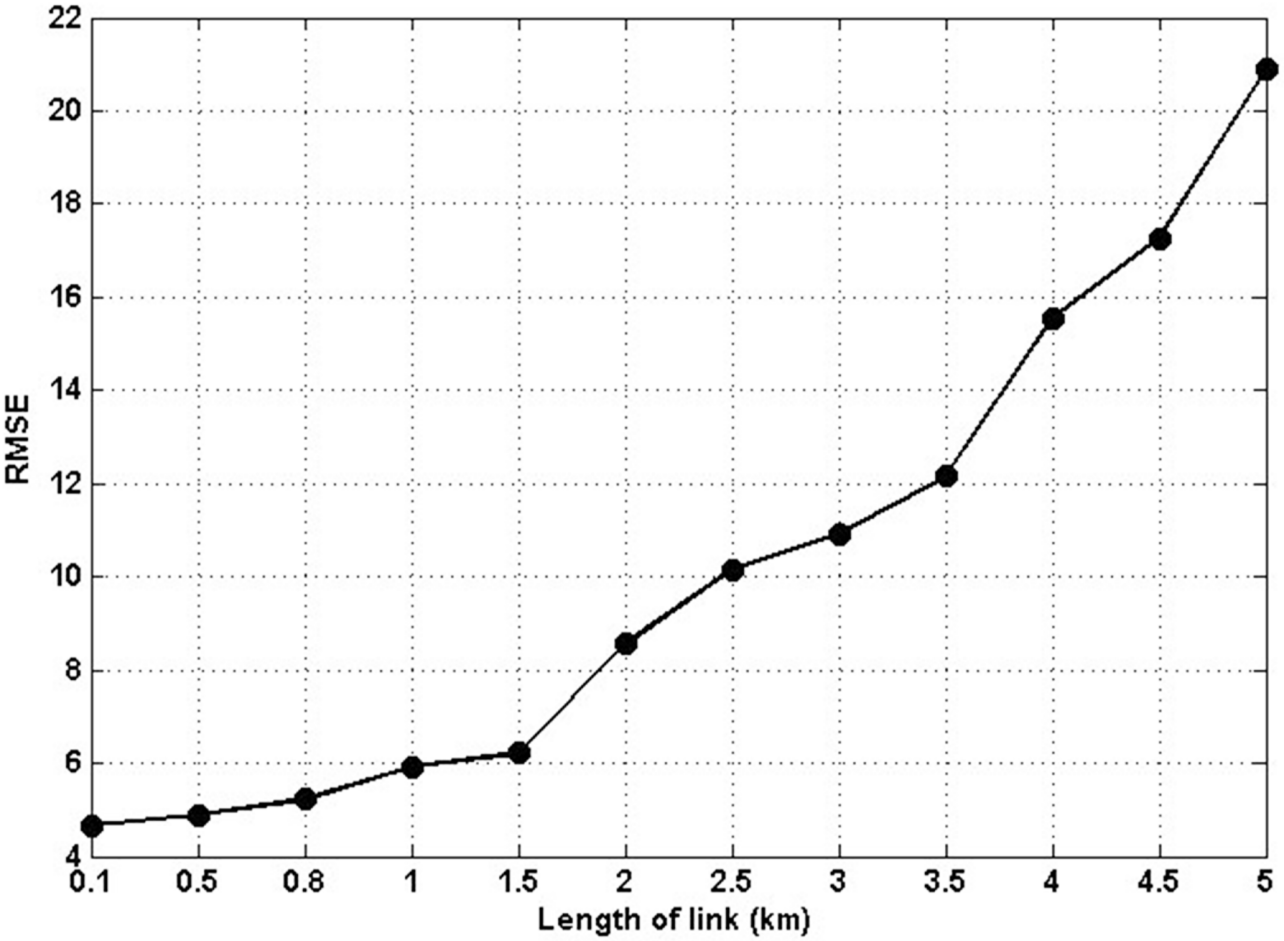
The influence of length of link to estimation accuracy.

We analyzed the influence of link length to estimation performance in the simulation environment, and the results were shown in Fig 9. The cluster number was 18, and the ratio *r* of training data used to calculate the initial parameters values in Eq (9) was set to 0.5. The data were also collected at 1-min interval. The samples from the former two days were used as training dataset and the remainder was used as testing dataset. We used RMSE as evaluating index. With the increase of the length of link, the estimation accuracy decreases gradually as expected. Especially when the length is longer than 2km, the increasing rate of error becomes faster. The reason is that the fixed detection devices cannot detect the traffic flow information in the middle of link. For the link with small length, the sudden changes of traffic flow state, for example, traffic congestion or accident, can be detected through observations of traffic flow data on upstream and downstream. However, when the link is long, such as longer than 2km, if we only consider information from endpoint detectors, the travel time estimation will be high erroneous. Therefore, in the process of travel time estimation by using the fixed detection devices, it is necessary to select relatively short link as experimental subject. While taking into account the economic factor, the distance between detectors cannot be set too small. Another effective approach is to fuse data source from fixed detectors and mobile sensors (probe vehicle equipped with GPS device). The mobile detectors provide internal information of traffic flow in the link, by which the travel time estimation performance can be obviously improved. This is also our main researching direction in future.

**Fig 10:**
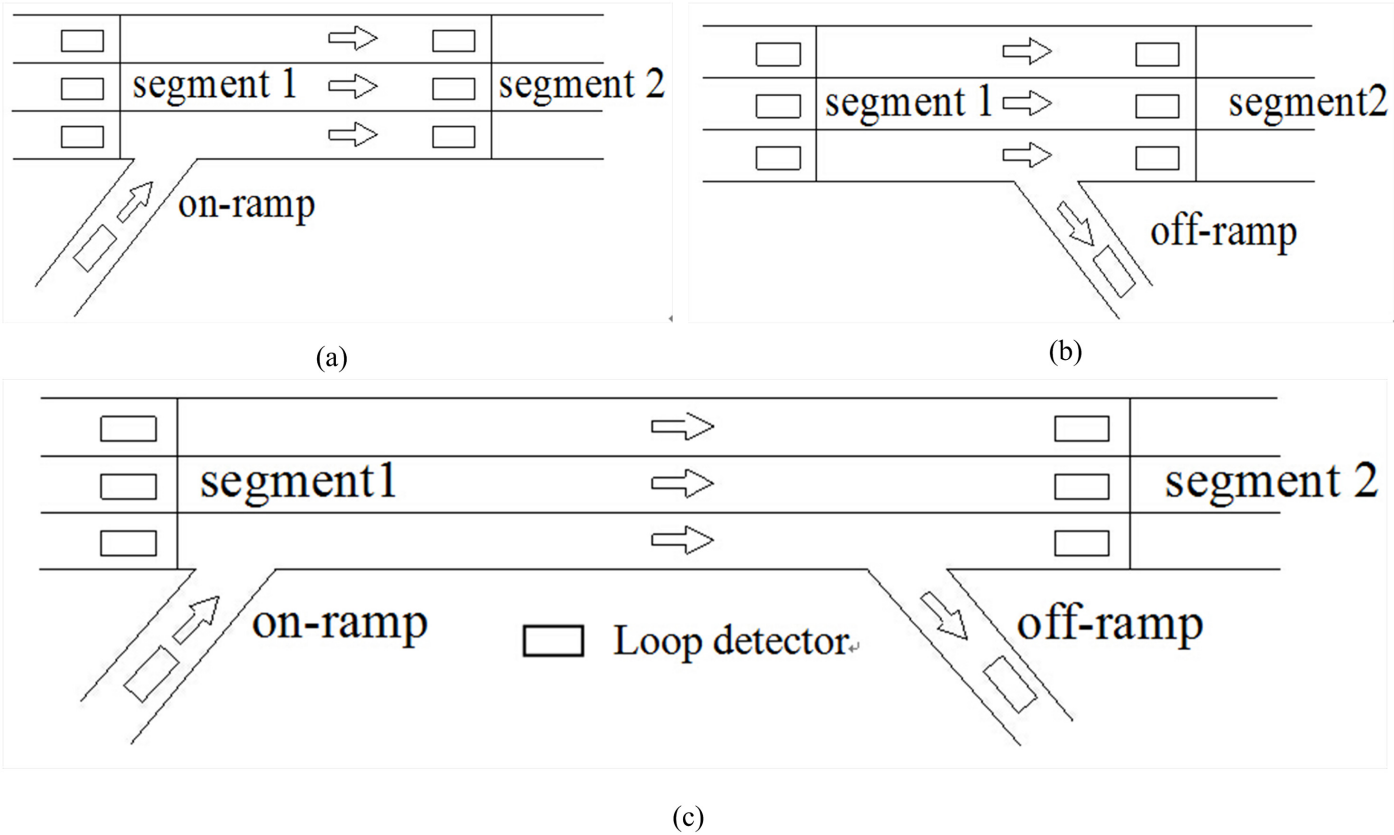
Links in application with ramps. (a) Case 1. (b) Case 2. (c) Case 3.

For the influence of ramps to performance, we applied the proposed model in three cases shown in Fig 10 to analyze the influence of ramp on travel time estimation performance. The three cases were expressed in simulation scenario. The first case shows a link with only on-ramp, the second case shows a link with an off-ramp, and the last one displays a link with both on-ramp and off-ramp. The cluster number, ratio *r* and data sample are same as above simulation process. We also use RMSE as evaluating index, and Table 10 shows the estimation results. As we can see, the estimation accuracy would decrease if we ignore the influence of ramps. In this study, the input samples of model include traffic flow data on upstream and downstream segments. For the case 1, the vehicles enter into link through on-ramp, and the vehicles exist from link through off-ramp in case 2. So, if the volumes of vehicle passing the on-ramp and off-ramp are ignored, samples on ramps are missing in input data. In the following process of model training and testing, the estimation accuracy will decline based on incomplete input samples.

**Table 10:** Simulation results for considering influence of entry and exit ramp.

	Upstream	Downstream	Length (km)	RMSE
Case 1	Segment1	Segment 2	0.8	3.72
	Segment1+on-ramp	Segment 2	0.8	2.99
Case 2	Segment1	Segment 2	0.8	3.49
	Segment1	Segment 2+ off-ramp	0.8	2.56
Case 3	Segment1	Segment 2	1.6	5.67
	Segment1+on-ramp	Segment 2+ off-ramp	1.6	4.53

## Conclusions

In this paper, we designed a new travel time estimator based on an evolving fuzzy neural network by using traffic flow data collected from existing loop detectors. In the network, traffic flow data (volume, occupancy, and speed) at points upstream and downstream of a given link were used as input variables, and link travel time was defined as the output variable. This EFNN model is based on the Takagi-Sugeno fuzzy inference system, in which *n* fuzzy rules are activated to calculate the output vectors for a given set of input vectors. In the training process, a K-means method was first used to cluster the input vectors and calculate the cluster centers. Then, the Gaussian-type fuzzy membership functions were designed to evaluate the membership degree of the input samples to each of the clusters. Finally, an improved weighted recursive LSE was used to optimize the parameters in the linear Takagi-Sugeno fuzzy inference functions. In the model verification process, the testing datasets collected in both actual and simulation scenarios were used to evaluate the performance of the proposed method in terms of three measurement criteria: MAE, RMSE, and MARE. The proposed model demonstrated its superiority when compared with some commonly used methods: MLR, IM, LM, NN, and CP.

Although our model provides a means to estimate the travel time data based on point infrastructure sensors, there are several limitations in the current work: (1) It does not consider data sources between the upstream and downstream points. The traffic flow data at upstream and downstream points can only reflect the in- and out-flow patterns on a link. We cannot obtain any information about the flows at intermediate locations on a given link. Thus, fusing data sources, that is to say combining data from detectors located at intermediate locations along links with data from the upstream and downstream detectors, would definitely improve the estimation accuracy of the proposed model. (2) This work only solves travel time estimation in freeway. However, in arterial roads, as travel times are influenced by intersections and traffic signals. The models used for travel time estimation in urban road networks should consider more information, such as the impacts of traffic signals, queue length, mid-link sources of delay, and turning movements. Thus, we will expand application of the proposed model in future research. (3) In the current work, we only discuss the influence of the link length to the travel time estimation accuracy. How to improve estimation performance of long link is also a key issue in actual applications. Fusing the floating car information is a possible solution in future study. (4)The presented model only performs one-step-ahead estimation of travel time. In future work, it would be interesting to study how to accurately estimate the multi-step-ahead values based on the same input vectors.

## Supporting Information

S1 File Loop detectors data. Data source collected from loop detectors in 15 days.
(XLSX)

S2 File Travel time data. Data source collected from Here in 15 days.
(XLSX)
